# Normal-to-Supercooled Liquid Transition in Molecular Glass-Formers: A Hidden Structural Transformation Fuelled by Conformational Interconversion

**DOI:** 10.1021/acs.jpcb.4c01025

**Published:** 2024-05-10

**Authors:** Andrzej Nowok, Joanna Grelska, Mateusz Dulski, Anna Z. Szeremeta, Kinga Łucak, Karolina Jurkiewicz, Hubert Hellwig, Sebastian Pawlus

**Affiliations:** † Department of Experimental Physics, Wrocław University of Science and Technology, Wybrzeże Stanisława Wyspiańskiego 27, 50-370 Wrocław, Poland; ‡ Laboratoire National des Champs Magnétiques Intenses, EMFL, CNRS UPR 3228, Université Toulouse, Université Toulouse 3, INSA-T, 31400 Toulouse,France; § August Chełkowski Institute of Physics, University of Silesia in Katowice, 75 Pułku Piechoty 1, 41-500 Chorzów, Poland; ∥ Faculty of Science and Technology, Institute of Materials Engineering, University of Silesia in Katowice, 75 Pułku Piechoty 1A, 41-500 Chorzów, Poland; ⊥ Center for Integrated Technology and Organic Synthesis (CiTOS), MolSys Research Unit, University of Liège, B6a, Room 3/19, Allée du Six Août 13, 4000 Liège, Belgium

## Abstract

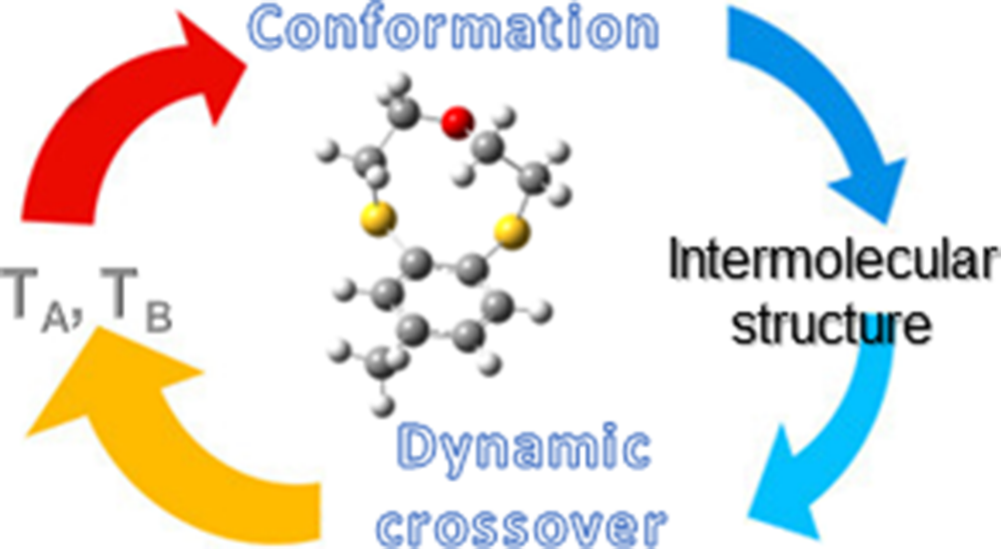

Molecular dynamics and transport coefficients change significantly around the so-called Arrhenius crossover in glass-forming systems. In this article, we revisit the dynamic processes occurring in a glass-forming macrocyclic crown thiaether **MeBzS**_**2**_**O** above its glass transition, revealing two crossover temperatures: *T*_B_ at 309 and *T*_A_ at 333 K. We identify the second one as the Arrhenius crossover that is closely related to the normal-to-supercooled liquid transition in this compound. We show that the transformation occurring at this point goes far beyond molecular dynamics (where the temperature dependence of structural relaxation times changes its character from activation-like to super-Arrhenius), being reflected also in the internal structure and diffraction pattern. In this respect, we found a twofold local organization of the nearest-neighbor molecules via weak van der Waals forces, without the formation of any medium-range order or mesophases. The nearest surrounding of each molecule evolves structurally in time due to the ongoing fast conformational changes. We identify several conformers of **MeBzS**_**2**_**O**, demonstrating that its lowest-energy conformation is preferred mainly at lower temperatures, i.e., in the supercooled liquid state. Its increased prevalence modifies locally the short-range intermolecular order and promotes vitrification. Consequently, we indicate that the Arrhenius transition is fuelled rather by conformational changes in this glass-forming macrocyclic crown thiaether, which is a different scenario from the so-far existing concepts. Our studies combine broadband dielectric spectroscopy (BDS), X-ray diffraction, Fourier transform infrared (FTIR) spectroscopy, molecular dynamics (MD) simulations, and density functional theory (DFT) calculations.

## 1 Introduction

For centuries, supercooled liquids have played a vital role in science, industry, and the day-to-day life of human beings.^1−3^ They develop when their chemical components fail to create crystallization nuclei below the freezing point. Consequently, the formation of supercooled liquids is often related to the manufacturing of glasses (including the molecular ones) via the vitrification process, in which progressive lowering of the temperature results in dramatic slowing down of molecular motions from nanoseconds to hundreds of seconds around the glass-transition temperature (*T*_g_).^1,4,5^ This process takes place in both the nonergodic and ergodic dynamic domains of the ultraviscous liquid, which intersect at the crossover temperature *T*_*B*_.^6,7^ However, well above *T*_g_ and *T*_B_, there is another characteristic point at which intermolecular organization, molecular motion, and transport coefficients (e.g., diffusivity, viscosity) undergo significant changes.^8−10^ These transformations delineate collectively the inflection point which is often referred to as the Arrhenius crossover.^11−15^

In general, molecular dynamics in normal and supercooled liquids can be monitored by broadband dielectric spectroscopy.^4^ This technique probes the reorientation of molecular dipole moments in response to the applied electric field, the collective motion of which gives rise to the structural relaxation process (α relaxation).^4^ The Arrhenius transition occurs at the crossover temperature *T*_A_ when the related structural relaxation time τ_α_ is roughly 60 ps.^16^ At this point, the temperature dependence τ_α_(*T*) shifts its pattern from a super-Arrhenius (typical for the ultraviscous regime) to what is often, but not always, the Arrhenius-like one above *T*_A_.^17,18^ The exceptional behavior was reported for, e.g., propylene carbonate or α-picoline.^10,18^ Apart from that, numerous additional discontinuous changes at *T*_A_ have been documented, e.g., changes in the inhomogeneous broadening of Raman lines,^19,20^ or additional contribution to the Landau–Placzek ratio.^21,22^ Finally, some universal relationships have been identified between *T*_A_, *T*_g_, and melting point *T*_m_. For example, the ratio *T*_A_/*T*_g_ is roughly 2 for all metallic glass-forming systems, falls within the range of 1.4–2.1 for molecular glass-formers, or spans between 1.6 and 4 for network liquids.^11,23^ It is also common to observe *T*_A_/*T*_m_ > 1 for good glass-formers.^24^ Despite the immense knowledge, the origin and the character (continuous, discontinuous) of the Arrhenius transition remain elusive.^17^ According to several models, temperatures exceeding *T*_A_ facilitate the relatively independent movement of particles, eliminating the necessity for a collective rearrangement of their local environment.^11,25^ In turn, due to reduced mobility, there is a requirement for the collective restructuring of molecules over a larger length scale to enable molecular motion within the ultraviscous, densely packed liquids.^11,25^ In these models, the emergence of the Arrhenius crossover is, thus, connected with the formation of ‘locally favored structures’, where the nearest-neighbor molecules are strongly coupled according to the scheme that is incompatible with any long-range order.^26−28^ Consequently, their formation is expected to substantially reduce the crystallization propensity, leading also to the appearance of an effective molecular field and growing inhomogeneities with a lowering of the temperature.^26−28^ Unfortunately, the concepts neglect molecular flexibility and related intramolecular dynamics, which are also crucial for intermolecular organization.^29^

In this article, we re-investigate the dynamics and intermolecular organization of a simple van der Waals crown-like glass-former: 2,3-(4′-methylbenzo)-1,4-dithia-7-oxacyclononane (abbreviated as **MeBzS**_**2**_**O**). We observe fluctuations of the local, nearest-neighbor structure between two possible arrangements of molecules, and indicate conformational interconversion as a main driving force for the ongoing Arrhenius crossover. Consequently, we put forward a different concept of processes occurring around *T*_A_ for this compound. Finally, we analyze the versatile impact of the normal-to-supercooled liquid transformation, showing that it goes far beyond molecular dynamics. Our studies are based on the combination of broadband dielectric spectroscopy (BDS), Fourier transform infrared spectroscopy (FTIR), X-ray diffraction, quantum density functional theory (DFT) calculations, and molecular dynamics (MD) simulation.

## 2 Materials and Methods

### 2.1 Materials

**Figure 1:**
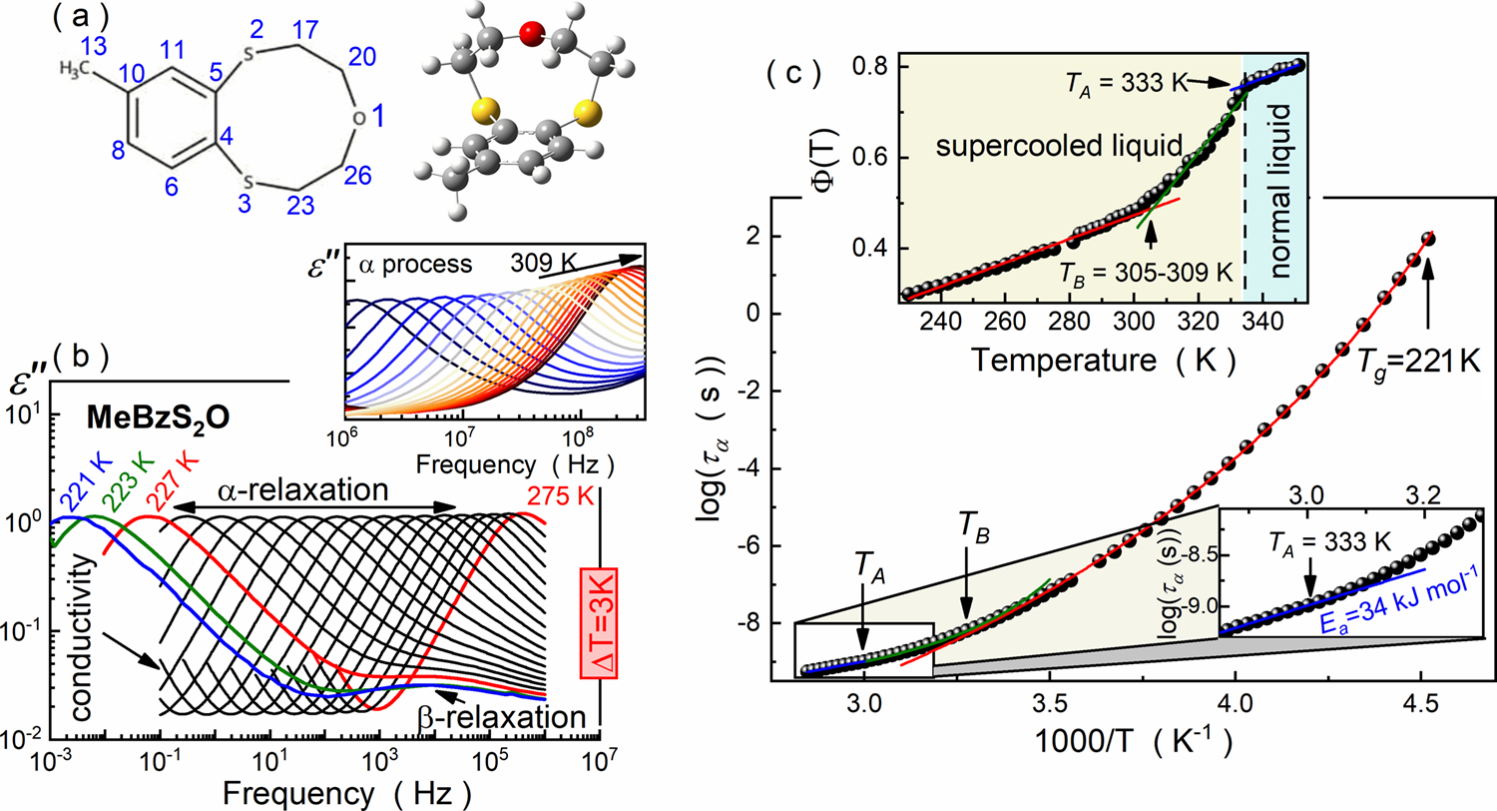
(a) Chemical structure of **MeBzS**_**2**_**O** with adopted atom numbering. (b) Selected ε″(*f*) measured for **MeBzS**_**2**_**O** in its supercooled liquid state between 221 and 275 K. The inset portrays high-frequency ε″(*f*) spectra collected in the range of 281–353 K with ongoing changes in the amplitude of α-relaxation loss peaks. (c) Temperature dependence of τ_α_ with marked glass transition. The upper inset shows the temperature dependence of Φ(*T*) with marked dynamic crossovers at *T*_A_ and *T*_B_. The lower inset portrays the transformation of the τ_α_(*T*) curve character from super-Arrhenius to Arrhenius-like during the normal-to-supercooled liquid transition.

The object of this research, **MeBzS**_**2**_**O**, has been identified as a moderately fragile crown-like glass-former characterized by *T*_m_ and *T*_g_ equal to 331 and 221–224 K (depending on the experimental method), respectively.^30^ It contains an aromatic benzene moiety and a heterocyclic ring with one oxygen and two sulfur atoms connected by ethylene −CH_2_–CH_2_– bridges (see Figure 1a). This chemical compound is commercially unavailable, and its synthesis, purification method, and purity have been discussed by us previously.^30^

### 2.2 Broadband Dielectric Spectroscopy

**MeBzS**_**2**_**O** was subjected to dielectric studies under ambient pressure conditions in the frequency range of 10^–3^–10^9^ Hz. Dielectric measurements between 10^–3^ and 10^6^ Hz were performed utilizing a Novocontrol GMBH Alfa impedance analyzer and a parallel-plate capacitor with a fixed distance between its electrodes equal to 0.1 mm. The electrodes were made of stainless steel, had a diameter of 10 mm, and were separated by two silica fibers. In contrast, higher-frequency dielectric measurements (between 10^6^ and 10^9^ Hz) were performed using an Agilent 4291B impedance analyzer connected with a Novocontrol GmbH system, and a parallel-plate capacitor with gold-plated electrodes. In this configuration, the electrodes had a diameter of 5 mm and the distance between them was kept at 0.06 mm by means of two silica fibers. Both capacitors were filled with the investigated material (**MeBzS**_**2**_**O**) after its prior melting. The broadband dielectric measurements were performed every 2 or 3 K at a wide temperature range (221–353 K) covering the normal liquid and supercooled liquid regimes. Each dielectric spectrum was collected after prior temperature stabilization. The temperature was precisely controlled by a Novocontrol Quattro system and stabilized using nitrogen gas with a precision exceeding 0.1 K.

### 2.3 X-ray Diffraction

Rigaku-Denki S/MAX RAPID II-R diffractometer equipped with a two-dimensional image plate detector and an Ag rotating anode was used to obtain X-ray diffraction patterns. The wavelength of the incident beam was 0.5608 Å. The temperature was controlled by Oxford Cryostream Plus and Compact Cooler. Samples were measured at ambient pressure between 245 and 370 K. The empty capillary was used for background measurement. Diffraction patterns were then corrected, normalized, and transformed into structure factors. In order to obtain amplitudes of the main peaks in structure factors, Voigt functions were used to fit the data at the same offset in the same scattering vector *Q* range covering the first and second diffraction maxima.

### 2.4 DFT Calculations

Density functional theory (DFT)^31,32^ at a hybrid B3PW91 level of theory^33^ and the 6-311++G(d,p) basis set^34−36^ was employed to investigate conformational diversity in **MeBzS**_**2**_**O**. The choice of this specific methodology and basis set was justified by previously established satisfactory agreement between experimental data and theoretical predictions of the energy barrier for intramolecular conformational changes in this compound.^30^ For the same reason, the input geometry for all calculations was based on the previously reported structure (conformer S_3_).^30^ In the initial step of our study, we subjected conformation S_3_ to geometry optimization. Subsequently, a comprehensive conformational analysis was conducted with a ± 5° step size for all dihedral angles within its heterocycle ring. This process involved monitoring changes in energy while altering a single dihedral angle. As a result, we identified other energetically favored structures and the most plausible interconversion paths between them. Among the conformers found through this analysis were conformations S_1_ and S_2_, which emerged as the predominant conformations in MD simulations. Therefore, we extended our analysis to these geometries, exploring possible conformational transformations by systematically varying each dihedral angle in their heterocyclic ring, again with a ± 5° step size. All DFT computations were executed in the Gaussian 16, Revision C.01 software package.^37^

### 2.5 MD Simulations

Molecular dynamics simulations were conducted in the GROMACS 2022 package.^38−40^ The topology files were created in the Antechamber module.^41^ The GAFF^42^ force field dedicated to organic compounds with aromatic rings was used. The simulation box consisted of 1000 randomly distributed molecules reproduced from one molecule of conformer S_3_ optimized previously by DFT calculations. NPT ensemble was used with Nose-Hoover temperature coupling (time constant 0.1 ps), and MTTK pressure coupling (time constant 1 ps) was kept at 1 bar pressure. The simulations were conducted in the cooling regime from 365 to 295 K for 5 ns simulation time at each step. The trajectories were collected from the last 4 ns of each step. Based on them, structure factors were calculated using TRAVIS software,^43−45^ and dihedral angle distributions were calculated using the GROMACS subprogram *gmx angle*.

### 2.6 FTIR Measurements

Temperature-dependent FTIR measurements of **MeBzS**_**2**_**O** were conducted in absorbance mode on the amorphous sample within the temperature range of 373 and 173 K. For this reason, the investigated material was initially heated above its melting point (*T*_m_ = 331 K), placed between CaF_2_ glass plates separated by 1 μm while hot, and mounted in the measurement setup. Measurements were performed during the cooling cycle at intervals of 10 K to capture the temperature-induced changes effectively. The temperature step size was chosen so that the sample did not crystallize throughout the experiment. Spectra were collected in the 950–4000 cm^–1^ range, accumulating 16 scans with a spectral resolution of 4 cm^–1^. Postprocessing analysis encompassed baseline correction, as well as the removal of water and carbon dioxide.

## 3 Results and Discussion

The ambient-pressure dielectric properties of **MeBzS**_**2**_**O** below its glass-transition temperature (*T*_g_ = 221 K) have already been extensively examined.^30^ At this temperature range, the dielectric loss spectra are dominated by two secondary relaxation processes that obey the Arrhenius law with the activation energy of 32 and 19 kJ/mol, respectively.^30^ Therefore, this article focuses on the relaxation dynamics of **MeBzS**_**2**_**O** only above its *T*_g_, i.e., in the supercooled- and normal-liquid regimes.

Figure 1b portrays exemplary frequency-dependent dielectric loss spectra ε*″*(*f*) measured in a broad frequency range (10^–3^–10^9^ Hz) at various temperature conditions. Two relaxations are apparent in the vicinity of *T*_g_. Following previous reports on this compound, the less intense process is a secondary β-relaxation.^30^ In turn, the dominating one is a structural α-relaxation, which originates from the cooperative motion of molecules in the liquid.^30^ As temperature increases, the separation between both relaxations diminishes, eventually resulting in their merging into a single process. Such a phenomenon can induce significant changes in the amplitude of the relaxation loss peaks (and the corresponding maximum value of dielectric losses, ε*″*_max_) in molecular glass formers. For example, the merging of α and β processes in decahydroisoquinoline nearly doubles ε*″*_max_ at ambient pressure, raising it from approximately 0.25 around *T*_g_ to 0.45 at 208 K.^46^ In contrast, such a phenomenon has a marginal impact on the ε″_max_ value in the studied **MeBzS**_**2**_**O**, which experiences only a slight increase from the merging point up to roughly 309 K. Unexpectedly, a stepwise significant increase in the α-relaxation amplitude occurs above this temperature threshold (see inset in Figure 1b).

To get a deeper insight into the underlying mechanism, we examine the temperature-induced shift of the α-relaxation. For this purpose, we parametrize the α-process in the dielectric spectra by a Havriliak–Negami function with an added dc-conductivity term:
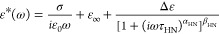
1where σ denotes the dc-conductivity of the material, ε_0_ is the dielectric constant of vacuum, ω is the angular frequency, ε_∞_ is the high-frequency limit of dielectric permittivity, Δε is dielectric strength, τ_HN_ denotes the so-called Havriliak–Negami relaxation time, and α_HN_, β_HN_ are the shape parameters describing the frequency dispersion of the loss peak.^47^ Further details of the fitting methodology are provided in Supporting Information. The structural relaxation times, τ_α_, that characterize the temperature-induced shifting of the α-relaxation losses in the ε″(*f*) spectra (), are then calculated as^4^
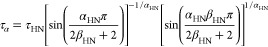
2

Typically, for molecular glass-formers,^1,4^ the temperature changes in τ_α_ exhibit a super-Arrhenius character in the vicinity of *T*_g_ that can be well described by a VFT equation:
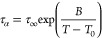
3where τ_∞_ is a pre-exponential factor, *B* is a material constant, and *T*_0_ is the so-called ideal glass temperature (Figure 1c).^48−50^ However, for **MeBzS**_**2**_**O**, a single VFT equation does not satisfactorily capture the temperature dependence of τ_α_ that span over 10 decades. In order to dissect the dynamic crossovers in the relaxation dynamics of **MeBzS**_**2**_**O** in its liquid phase, we follow the methodology proposed by Stickel et al.^51^ and calculate the quantity Φ(*T*) associated with the derivative of structural relaxation with respect to temperature:
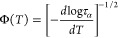
4

Such an approach is distortion-sensitive and allows linearizing the most popular Arrhenius and VFT equations according to the following formulas:
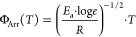
5
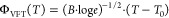
6

The temperature dependence of the quantity Φ is visualized in the upper inset in Figure 1c and reveals two dynamic crossovers at roughly *T*_B_ = 305–309 K and *T*_A_ = 333 K. The first one is related to a crossover between two super-Arrhenius dependencies of τ_α_, which can be well described by VFT equations with parameters from Table 1 (see red and green lines in Figure 1c). As the ratio *T*_B_/*T*_g_ is close to 1.3 (*T*_B_/*T*_g_ = 1.38), we identify *T*_B_ as a dynamic crossover temperature between low-temperature nonergodic and high-temperature ergodic domains in the ultraviscous regime.^5^ Notably, this transformation exhibits a diffuse character for **MeBzS**_**2**_**O**, and the ergodic region resembles rather a transient region between high-temperature normal liquid and the near-*T*_g_ supercooled liquid. In turn, we identify the *T*_A_ as a hallmark of the Arrhenius transition, in which the temperature dependence of τ_α_ changes its pattern from super-Arrhenius to Arrhenius-like with an apparent activation energy of *E*_a_ = 34 ± 1 kJ mol^–1^ (c.f. lower inset in Figure 1c). This transition occurs near the melting point (*T*_m_ = 331 K) and, thus, can be referred to as a signature of the normal-to-supercooled liquid transition. According to the literature, the emergence of this transformation should be connected with the onset of forming ‘locally favored structures’.^26−28^ In order to verify this hypothesis, further X-ray scattering (diffraction) studies supported by molecular dynamics and quantum DFT modeling are performed.

**Table 1:** Parameters Describing Temperature Dependence of τ_α_ in **MeBzS**_**2**_**O**

temperature range	fitting curve	*T*_0_ (K)	*B* (K)	log(τ_0_ (s))	*E*_a_ (kJ/mol)
*T* < *T*_B_	VFT	161 ± 1	2240 ± 50	–15.6 ± 0.1	
*T*_B_ < *T* < *T*_A_	VFT	252 ± 2	279 ± 15	–10.5 ± 0.1	
*T* > *T*_A_	Arrhenius			–14.2 ± 0.05	34 ± 1

**Figure 2:**
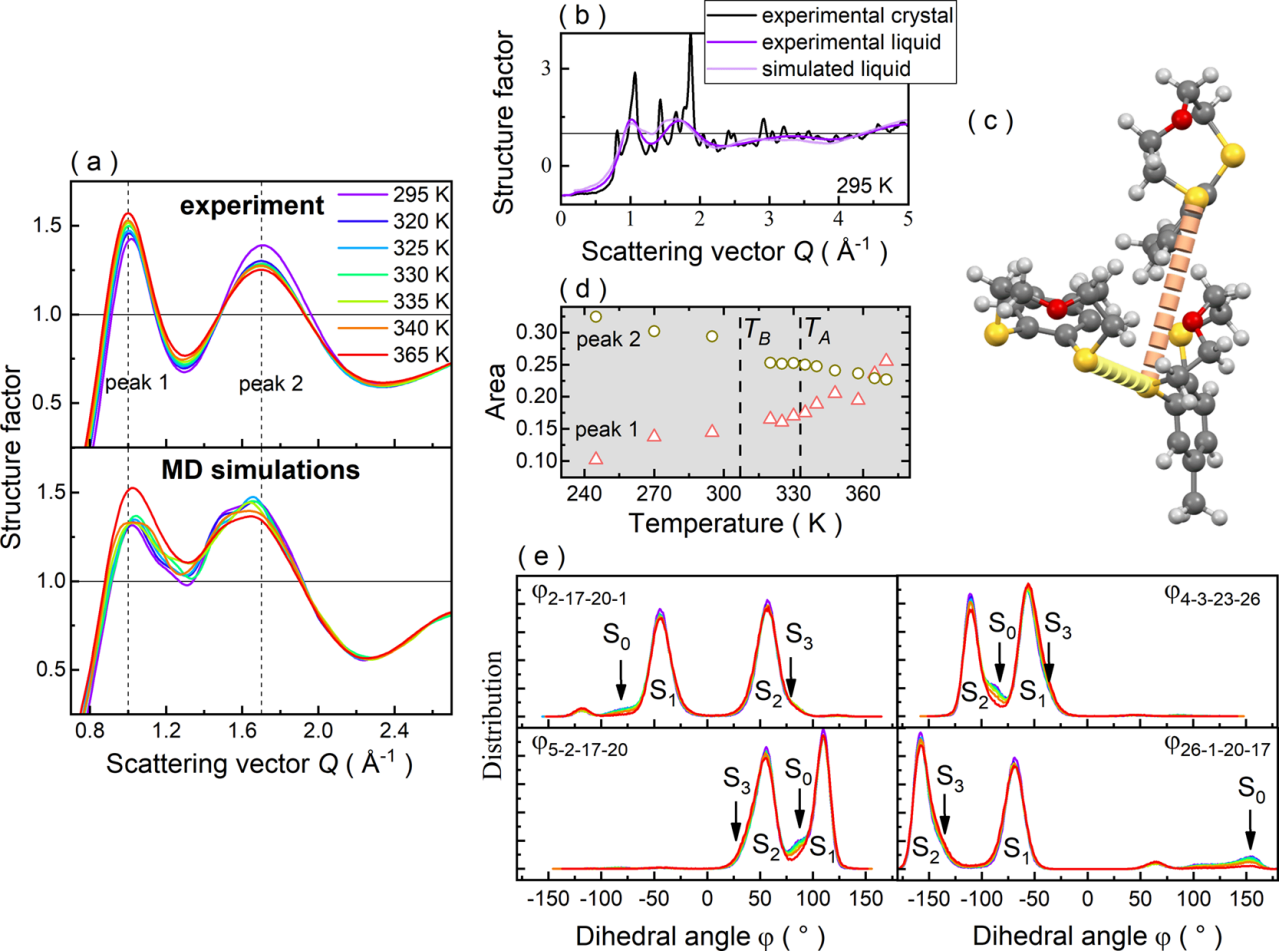
(a) Experimental and simulated structure factors of **MeBzS**_**2**_**O** at temperatures ranging between 295 and 365 K. (b) Structure factor for crystalline sample measured at 295 K compared with the structure factors for liquid phase derived from experiment and MD simulations. (c) Exemplary nearest neighbor arrangements of molecules with marked characteristic intermolecular distances. (d) Changes in the amplitudes of two main diffraction maxima derived from experimental data with marked crossover temperatures *T*_B_ and *T*_A_. (e) Dihedral angle distributions obtained from MD simulations. The peaks at specific angles are assigned the appropriate conformations S_0_, S_1_, S_2_, S_3_.

In the diffraction pattern of **MeBzS**_**2**_**O**—Figure 2a, one can notice two characteristic maxima in the positions of *Q* ∼ 1 and ∼1.7 Å^–1^, which correspond to the positions of the most intense Bragg peaks of the crystal phase (Figure 2b). These positions reflect the real spaces periodicities of ∼6.3 and ∼3.7 Å, respectively, and arise due to two types of the nearest neighbor arrangements of molecules (see Figure 2c). The behavior of the two diffraction maxima as a function of temperature is unusual. The amplitude of the first peak increases with increasing temperature, and inversely—the second maximum decreases (c.f. Figure 2a,d and Figure S3 in SI). However, the slopes of these trends change slightly around *T*_A_ and *T*_B_ temperatures, where also crossovers in molecular dynamics were revealed. Moreover, the transformation within the two main diffraction maxima indicates the ongoing temperature-induced transition between two preferred molecular alignments, which are further better depicted based on the MD model.

**Figure 3:**
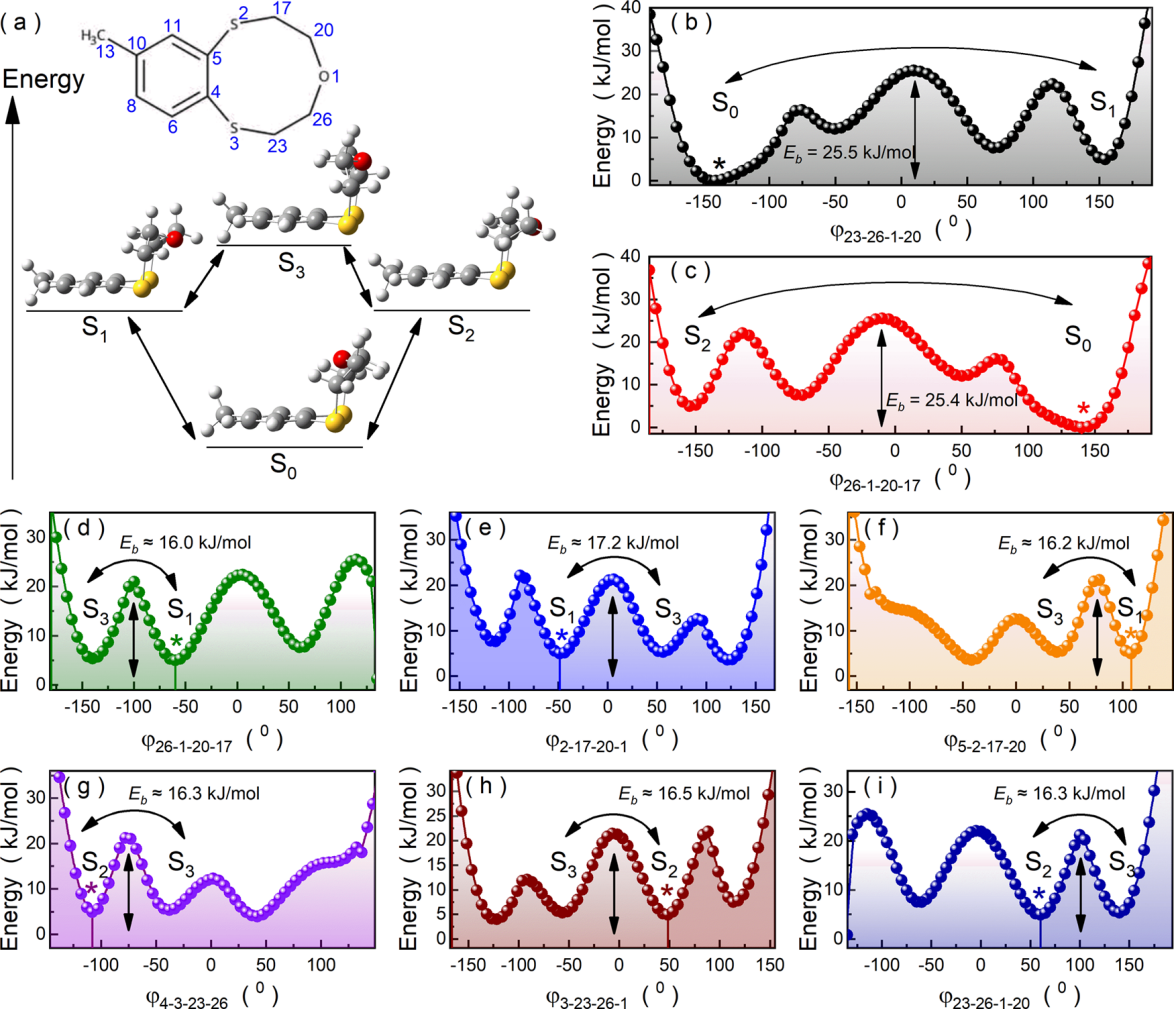
(a) Numeration of non-hydrogen atoms in the molecule of **MeBzS**_**2**_**O** and the geometries of its four conformers S_0_, S_1_, S_2_, S_3_. (b) Mutual interconversion between conformers S_0_ and S_1_ achieved by alteration in dihedral angle φ_23–26–1–20_. (c) Changes in potential energy while modifying the value of dihedral angle φ_26–1–20–17_. Variation in potential energy during transition between conformers S_3_ and S_1_ induced by changing the dihedral angle φ_26–1–20–17_ (d), φ_2–17–20–1_ (e), and φ_5–2–17–20_ (f). Variation in potential energy during the transition between conformers S_3_ and S_2_ induced by changing the dihedral angle φ_4–3–23–26_ (g), φ_3–23–26–1_ (h), and φ_23–26–1–20_ (i).

MD-derived structure factors are presented in the lower panel of Figure 2a. They show good compliance with the experimental results presented in the upper panel. Based on the MD-optimized systems, the dihedral angle distributions of molecules were calculated. From the dihedral angle distributions in Figure 2e, one can deduce various conformations coexisting with each other. We have identified four conformers of **MeBzS**_**2**_**O**: S_0_, S_1_, S_2_, and S_3_. As presented in Figure 3a, each contains two exodentate sulfur atoms, the lone pairs of which face away from the aromatic ring. Moreover, they all exhibit an energetically beneficial staggered conformation of the hydrogen atoms in both ethylene bridges −CH_2_–CH_2_–. The defining characteristics of conformers S_1_ and S_2_ include an asymmetric heterocyclic ring with its oxygen atom oriented in the opposite direction to the aromatic ring. The entire molecules of **MeBzS**_**2**_**O** in geometries S_0_ and S_3_ are also deprived of symmetry elements due to the presence of the methyl group −CH_3_. Nevertheless, they possess a symmetric heterocyclic ring (a mirror plane can be discerned considering only this chemical moiety). In conformer S_3_, the heterocyclic oxygen atom points away from the benzene ring, while in conformer S_0_, it is directed toward the aromatic group. Detailed data about the geometry of conformers S_0_–S_3_ are presented in Table S1 in Supporting Information.

Conformers S_1_ and S_2_ are the most abundant in the normal and supercooled liquids, independently of the temperature conditions (see Figure 2e). In turn, conformers S_0_ and S_3_ occur in smaller fractions. The temperature influence on the conformational distribution is evident: there is more conformational variety at low temperatures and, reversely, less variety of conformations at high temperatures where conformer S_0_ does not survive. These outcomes show that there are some considerable conformational changes induced by temperature in **MeBzS**_**2**_**O**. In order to elucidate this issue, we performed DFT calculations using a single-molecule approach.

According to the computations, conformer S_0_ has the lowest energy among the identified conformers S_0_–S_3_ (see Figure 3a). Specifically, conformers S_1_ and S_2_ are isoenergetic, whereas the potential energy of conformer S_0_ is lower by approximately 4.9 kJ/mol when compared to them. In turn, the difference between the potential energies of conformers S_3_ and S_0_ is equal to 5.4 kJ/mol. Consequently, the increasing prevalence of conformer S_0_ at lower temperatures aligns with the general principle that molecules tend to favor the ground state under such conditions. Its increased formation in the supercooled liquid state may also explain a considerably reduced tendency toward the crystallization of **MeBzS**_**2**_**O**, but further studies are required to confirm this hypothesis. It is important to note that conformers S_0_, S_1_, S_2_, and S_3_ can readily interconvert among themselves. This phenomenon is well illustrated by the potential energy curves, which portray the variations in the energy of the **MeBzS**_**2**_**O** molecule with respect to the S_0_ geometry as a dihedral angle changes. As presented in Figure 3b, a gradual increase in the dihedral angle φ_23–26–1–20_ from around −141° to approximately 152° allows transforming conformer S_0_ into S_1_ geometry. In turn, a stepwise reduction in the value of dihedral angle φ_26–1–20–17_ from roughly 140° to approximately −152° changes the geometry of conformer S_0_ to S_2_ (Figure 3c). Both interconversion paths lead via several higher-energy transient geometries, the structural characteristics of which are presented in SI materials. Some of these conformers have also been detected during the MD simulations.

Altering just one dihedral angle, a direct transformation of conformer S_1_ into S_3_ can be achieved in three ways: (1) decreasing the dihedral angle φ_26–1–20–17_ from ∼ −60° to ∼ −138° (Figure 3d), (2) by increasing the dihedral angle φ_2–17–20–1_ from ∼ −48° to ∼56° (Figure 3e), (3) by decreasing the dihedral angle φ_5–2–17–20_ from 107.5° to roughly 39° (Figure 3f). The energy barrier for each process takes the value of approximately 16–17 kJ/mol. However, these interconversion pathways are not equivalent, as can be observed when plotting the values of the dihedral angle φ_26–1–20–17_ against the dihedral angle φ_20–17–2–5_ (see Figure S8a in SI). A similar scenario occurs also for the conformational change between geometries S_2_ and S_3_. In this case, there are also three independent interconversion pathways, each characterized by the same energy barrier of ∼16.5 kJ/mol and the same transient geometry (see Figure S8b in SI). To shift the structure of conformer S_2_ into S_3_, one can either: (1) increase the value of dihedral angle φ_4–3–23–26_ from ∼ −108° up to ∼ −38° (Figure 3g), (2) decrease the value of dihedral angle φ_3–23–26–1_ from ∼48.5° to ∼ −56° (Figure 3h), (3) increase the value of dihedral angle φ_23–26–1–20_ from ∼60° to ∼138° (Figure 3i). Consequently, the results reveal at least nine distinct interconversion paths between conformers S_1_ and S_2_, all leading via state S_3_. Moreover, they suggest that all the conformers remain in thermal equilibrium, strongly affected by temperature. To validate the theoretical predictions, we reinvestigated **MeBzS**_**2**_**O** in its liquid and glassy states by means of FTIR spectroscopy.

**Figure 4:**
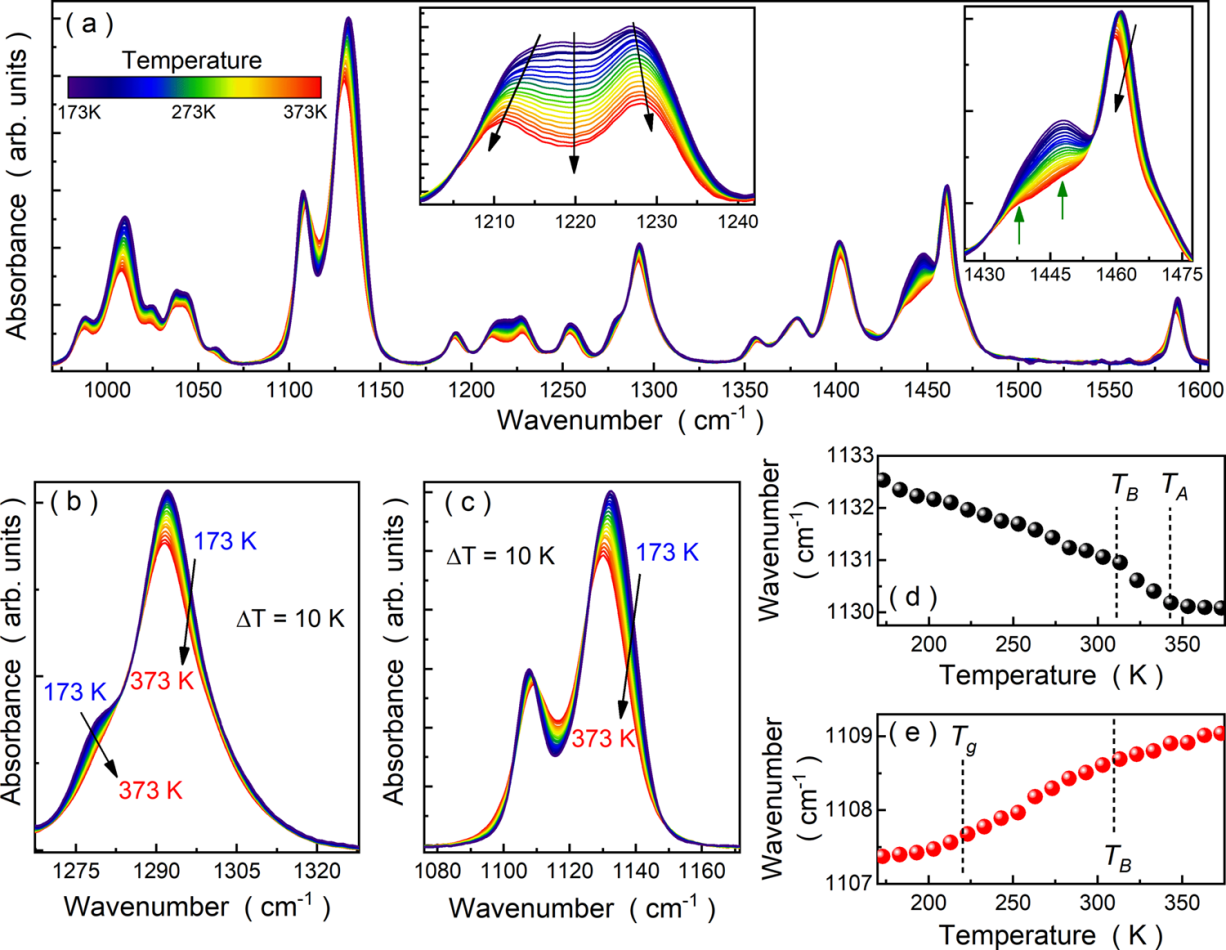
(a) FTIR spectra measured for **MeBzS**_**2**_**O** between 273 and 373 K and presented in the spectral range of 970–1605 cm^–1^. The middle and right insets illustrate changes in the line shape of bands between 1200 and 1240, and 1425–1477 cm^–1^. Line shape alterations observed during cooling for the characteristic bands between 1265–1330 cm^–1^ (b) and 1075–1170 cm^–1^ (c). Temperature-induced changes in the wavenumber of exemplary IR bands positioned at approximately 1131 cm^–1^ (d) and 1108 cm^–1^ (e) with marked glass-transition and crossover temperatures *T*_B_ and *T*_A_.

In this experiment, we systematically collected FTIR spectra at 10 K intervals while cooling from 373 to 173 K. Our primary focus was put on the fingerprint region extending below 1600 cm^–1^, where noteworthy temperature-induced changes were reported.^30^Figure 4a illustrates that, within this range, many bands are only slightly affected by temperature. Considerable alterations occur for bands positioned in-between 1075–1170, 1200–1240, 1265–1330, and 1425–1480 cm^–1^ (Figure 4a–c). For example, the bands at roughly 1210 and 1230 cm^–1^ at 373 K converge as the temperature decreases, shifting toward higher and lower wavenumbers, respectively (see left inset in Figure 4a). These temperature-induced spectral changes are coupled with variations in intensity. Additionally, a novel contribution emerges at lower temperatures around 1220 cm^–1^. Noteworthy changes in spectral line shape appear also in the 1425–1480 cm^–1^ range, where the band cantered at ∼1445 cm^–1^ becomes more pronounced as the temperature decreases, and the band around 1460 cm^–1^ shifts toward higher wavenumbers (right inset in Figure 4a). According to the literature, the enumerated bands are linked to diverse vibrational modes within the −CH_2_–CH_2_ bridges of the heterocyclic ring in **MeBzS**_**2**_**O**, as well as distortions within the ether -O- moiety.^30^ Hence, consistent with our predictions made by theory and MD simulations, the observed spectral changes point toward ongoing dynamical processes within the skeleton of **MeBzS**_**2**_**O**, indicating that its various conformers remain in temperature-dependent thermal equilibrium.

The conformational transformations leading to conformer S_0_ substantially reshape the **MeBzS**_**2**_**O** molecule, influencing the spatial distribution of its atomic charges. As a result, the emergence of conformer S_0_ at lower temperatures significantly perturbs the arrangement of neighboring molecules within its first coordination sphere (see SI for more information). This phenomenon is manifested in a direct correlation between the prevalence of conformer S_0_ and the intensities of the primary diffraction peaks (c.f., Figure 2a,b). Therefore, we recognize conformational changes in **MeBzS**_**2**_**O** as key factors fuelling the structural and dynamical transformations at temperatures *T*_A_ and *T*_B_. This conclusion is further supported by the correspondence between characteristic points *T*_A_ and *T*_B_ and noticeable alterations in line shape and band position in FTIR spectra (see Figure 4d,e). Consequently, we demonstrate that intramolecular dynamics not only underlies dielectric secondary relaxation processes in **MeBzS**_**2**_**O** but also controls its intermolecular arrangement, influencing the dynamics of entire molecules and contributing to the macroscopic Arrhenius crossover phenomenon. In this context, the behavior of **MeBzS**_**2**_**O** differs from that of other typical molecular glass-formers, such as phenolphthalein-dimethyl-ether (PDE), *o*-terphenyl (OTP), salol, or propylene carbonate (PC).^52,53^ In these systems, crossover phenomena (such as those related to *T*_B_) originate primarily from dramatically rising intermolecular cooperativity (many-body effects), as indicated by the Coupling Model and the absence of any crossover in the intermolecularly uncoupled (noncooperative) relaxation times τ_*0*_.^52,53^**MeBzS**_**2**_**O** can thus be regarded as an extreme case of pronounced conformational changes, highlighting that intramolecular degrees of freedom may also play a crucial role in comprehending the dynamics of liquids across a wide temperature range. Finally, our analysis suggests that the rising concentration of the less polar conformer S_0_ and the corresponding intermolecular reordering within its surrounding (first coordination sphere) may also constitute the physical origin of changes in the maximum value of dielectric losses (ε″_max_) around 309 K. The reason for this lies in the fact that the overall dielectric strength is proportional to the square of the permanent dipole moments that are responsible for the dipole density fluctuation.^54^

## 4 Conclusions

Molecular dynamics and transport coefficient change substantially in molecular glass-formers when their liquid phase becomes supercooled. This trend also occurs for the herein-studied compound, 2,3-(4′-methylbenzo)-1,4-dithia-7-oxacyclononane (**MeBzS2O**), which is a small thiacrown ether with a melting point (*T*_m_) of 331 K and glass-transition temperature (*T*_g_) of roughly 221 K. We uncover two crossover temperatures for this glass-former: *T*_B_ at 309 K and *T*_A_ at 333 K. The first one is a dynamic crossover temperature between low-temperature nonergodic and high-temperature ergodic domains in the ultraviscous regime, with the *T*_B_/*T*_g_ ratio close to 1.3 (*T*_B_/*T*_g_ = 1.38). At this point, the temperature dependence of structural relaxation times (τ_α_), which are associated with the motion of entire molecules, shifts between two different super-Arrhenius patterns. In turn, *T*_A_ is identified as the Arrhenius crossover closely related to the normal-to-supercooled liquid transition in **MeBzS**_**2**_**O**. Here, the temperature dependence of τ_α_ changes its pattern from super-Arrhenius to Arrhenius-like, with an apparent activation energy of *E*_a_ = 34 ± 1 kJ mol^–1^. However, the transformation at *T*_A_ extends beyond molecular dynamics, being reflected also in the internal structure and diffraction pattern. In this respect, we found a 2-fold organization of the nearest-neighbor molecules with periodicities around 6.3 and 3.7 Å in real space. The local short-range structures experience time- and temperature-dependent fluctuations due to the ongoing conformational changes of **MeBzS**_**2**_**O** molecules. Especially conformational transformations leading to the lowest-energy conformer S_0_ substantially reshape the **MeBzS**_**2**_**O** molecules, perturbing the nearest-neighbor intermolecular organization toward a denser packing. This process intensifies at lower temperatures, leading to a counterintuitive increase in the conformational diversity of **MeBzS**_**2**_**O** in its supercooled liquid phase. For example, the conformer S_0_ does not persist in the normal liquid (above *T*_m_), which is dominated by conformations with an asymmetrical heterocyclic ring. A direct correlation is also observed between the prevalence of conformer S_0_, the intensities of the primary diffraction peaks, and the emergence of the *T*_A_ and *T*_B_ points. Therefore, we recognize conformational changes in **MeBzS**_**2**_**O** as key factors driving the structural and dynamical transformations at temperatures *T*_A_ and *T*_B_. In essence, intramolecular dynamics not only underlies dielectric secondary relaxation processes in **MeBzS**_**2**_**O** but also governs its intermolecular arrangement, influencing the dynamics of entire molecules and fuelling the normal-to-supercooled liquid transition.

## Supporting Information Available

Additional information for dielectric data analysis, supporting information for MD simulations, and details about DFT-optimized geometries of conformers S_0_–S_3_ and their intramolecular rearrangement possibilities (PDF)
