# Supplementary material for: Normal-to-Supercooled Liquid Transition in Molecular Glass-Formers: A Hidden Structural Transformation Fuelled by Conformational Interconversion
Source: J Phys Chem B. 2024 May 10;128(20):5055–63. doi: 10.1021/acs.jpcb.4c01025 (PMC11129183; doi:10.1021/acs.jpcb.4c01025)
Supplement: Supplementary file 1 — jp4c01025_si_001.pdf [file jp4c01025_si_001.pdf]

Normal-To-Supercooled Liquid Transition in Molecular Glass-Formers: a Hidden Structural Transformation Fuelled by Conformational Interconversion

Supplementary Materials

Andrzej Nowok[a,b,*], Joanna Grelska[c], Mateusz Dulski[d], Anna Z. Szeremeta[c], Kinga Łucak[c], Karolina Jurkiewicz[c], Hubert Hellwig[e] and Sebastian Pawlus[c]

[a]*Department of Experimental Physics, Wrocław University of Science and Technology, Wybrzeże Stanisława Wyspiańskiego 27, 50-370 Wrocław, Poland*

[b]*Laboratoire National des Champs Magnétiques Intenses, EMFL, CNRS UPR 3228, Université Toulouse, Université Toulouse 3, INSA-T, Toulouse 31400, France*

[c]*August Chełkowski Institute of Physics, University of Silesia in Katowice, 75 Pułku Piechoty 1, 41-500 Chorzów, Poland*

[d]*Faculty of Science and Technology, Institute of Materials Engineering, University of Silesia in Katowice, 75 Pułku Piechoty 1A, 41-500 Chorzów, Poland*

[e]*Center for Integrated Technology and Organic Synthesis (CiTOS), MolSys Research Unit, University of Liège, B6a, Room 3/19, Allée du Six Août 13, 4000 Liège, Sart Tilman, Belgium*

*andrzej.nowok@pwr.edu.pl

1. Additional information for dielectric data analysis

In order to describe properly the entire frequency-dependent near-T_g dielectric spectra of **MeBzS$_2$O** (i.e., the real and imaginary parts of the complex dielectric permittivity), one should use the combination of the Cole-Cole formalism[1] and the Havriliak-Negami function[2]. In such methodology, the Cole-Cole function is used to parametrize the β relaxation process which contributes to ε' and ε'' in the high-frequency range. In turn, the most general Havriliak-Negami function is used to describe the α process due to its asymmetric shape. The addition of conductivity term is also required in a higher temperature range. Consequently, the fitting function takes the following form:

$$\varepsilon^*(\omega) = \frac{\sigma}{i\varepsilon_0\omega} + \varepsilon_\infty + \frac{\Delta\varepsilon_\alpha}{\left(1+(i\omega\tau_{HN})^{\alpha_{HN}}\right)^{\beta_{HN}}} + \frac{\Delta\varepsilon_\beta}{1+(i\omega\tau_{CC})^{\alpha_{CC}}}, \quad \text{(S1)}$$

where σ denotes the dc conductivity of the material, ε_0 is the dielectric constant of vacuum, ω is angular frequency, ε_∞ is the high-frequency limit of dielectric permittivity, $\Delta\varepsilon_\alpha$ is dielectric strength of the α process, τ_{HN} denotes the so-called Havriliak-Negami relaxation time, α_{HN}, β_{HN} are the shape parameters describing the frequency dispersion of the α process, $\Delta\varepsilon_\beta$ is dielectric strength of the β process, τ_{CC} denotes the so-called Cole-Cole relaxation time, and α_{CC} is parameter describing the symmetric broadening of the β process. As presented in Figure S1a,b, this model parametrize well the near-T_g dielectric spectra of **MeBzS$_2$O**. The structural relaxation times τ_α are then calculated based on the formula:

$$\tau_\alpha = \tau_{HN}\left[\sin\left(\frac{\alpha_{HN}\pi}{2\beta_{HN}+2}\right)\right]^{-\frac{1}{\alpha_{HN}}}\left[\sin\left(\frac{\alpha_{HN}\beta_{HN}\pi}{2\beta_{HN}+2}\right)\right]^{\frac{1}{\alpha_{HN}}}. \quad \text{(S2)}$$

[3]

In turn, the relaxation times τ_β are taken as $\tau_\beta = \tau_{CC}$.

However, we notice that comparable values of τ_α can be obtained by neglecting the contribution of the less-intense β-relaxation, and fitting the dielectric spectra only in the vicinity of the α relaxation peak with the Havriliak-Negami function with an added dc-conductivity term (c.f. Figure S2b and S2c). Such methodology allows also avoiding any model-dependent fitting procedure at higher temperatures, where the β and α processes overlap. Therefore, this procedure was implemented to determine the τ_α values for **MeBzS$_2$O** in this article.

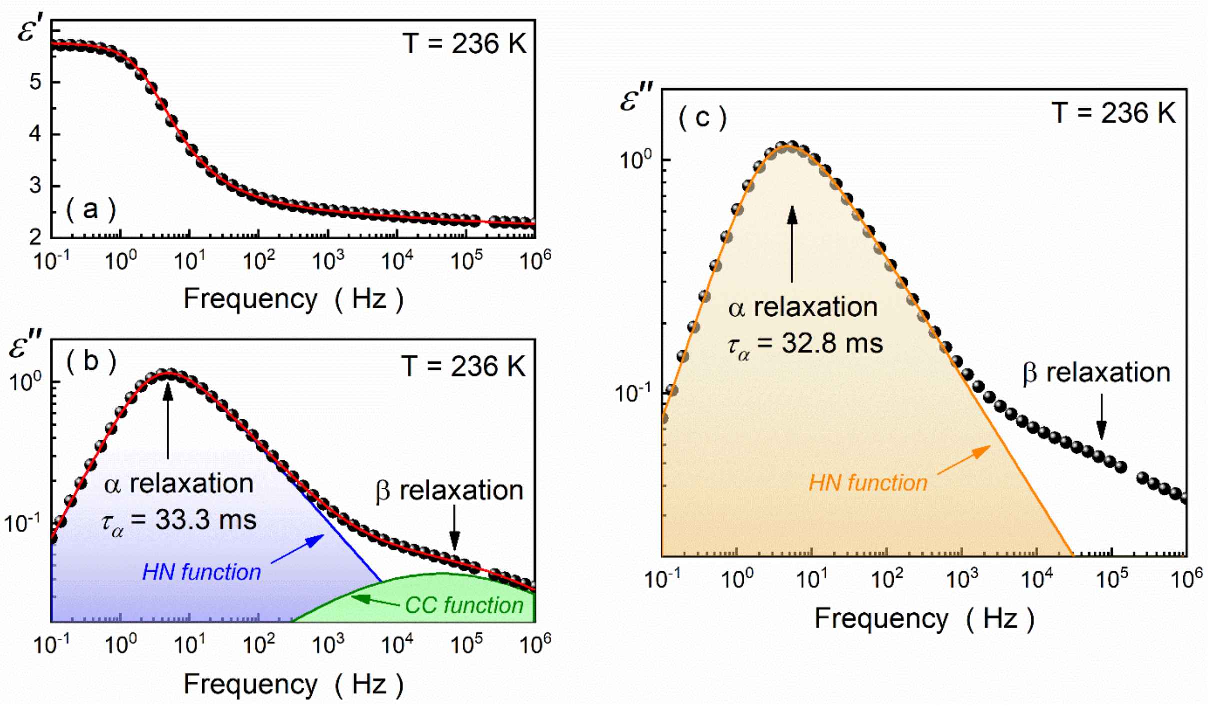

Figure S1. (a) Exemplary $\varepsilon'(f)$ spectrum collected for **MeBzS$_2$O** at 236 K fitted with a sum of the Havriliak-Negami and Cole-Cole functions. (b) Corresponding $\varepsilon''(f)$ spectrum described by the same combination of fitting functions. (c) Exemplary fitting of α relaxation peak by only a single Havriliak-Negami function.

In the frame of the derivative analysis of relaxation times proposed by Stickel *et al.*, we explored an alternative methodology based on the operator $\Phi(T^{-1}) = \left[-\frac{d\, log_{10}\tau_\alpha}{d(T^{-1})}\right]^{-1/2}$.[4] This approach transforms the Arrhenius and VFT equations into the following formulas:

$$\Phi_{Arr}(T^{-1}) = \left(\frac{E_a \cdot loge}{R}\right)^{-1/2}; \quad \text{(S3)}$$

$$\Phi_{VFT}(T^{-1}) = (B \cdot loge)^{-1/2} \cdot \left(1 - \frac{T_0}{T}\right). \quad \text{(S4)}$$

Hence, the $\phi(T^{-1})$ operator converts the Arrhenius dependence into a constant and linearizes the VFT curve on a 1/T (or 1000/T) scale. In the case of **MeBzS$_2$O**, $\phi(T^{-1})$ remains basically constant above 333 K, justifying the application of the Arrhenius law for fitting the $\tau_\alpha(T)$ dependence in this temperature range. The variable $\phi(T^{-1})$ below 333 K exhibiting a kink around 305-309 K indicates the necessity of using two independent VFT curves in this temperature regime.

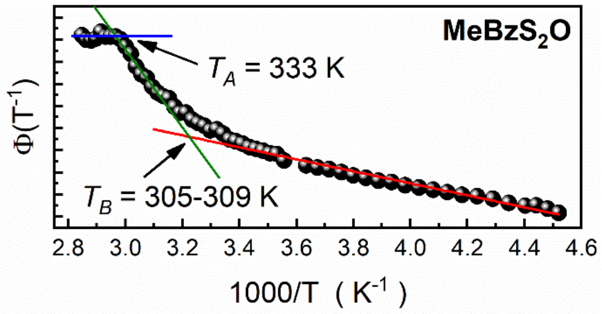

Figure S2. Temperature dependence of $\Phi(T^{-1})$ with marked dynamic crossovers at T_A and T_B.

2. Additional data for X-ray diffraction

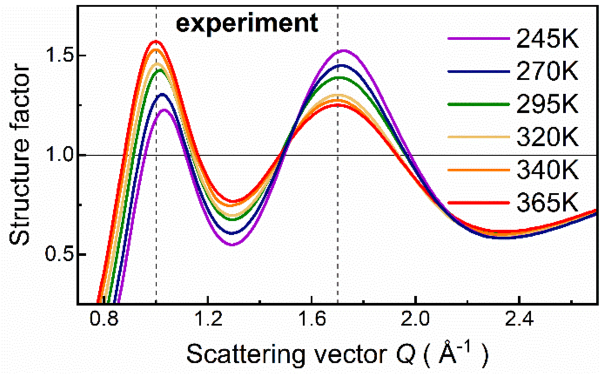

Figure S3. Experimental structure factors of **MeBzS$_2$O** at temperatures ranging between 245 K and 365 K presented with a step of roughly 25 K.

As presented in Figure S3, the amplitude of the first peak increases with increasing temperature, and inversely - the second maximum decreases. However, the slope of these changes switch around T_A temperature (i.e. 320-340 K), where also crossover in molecular dynamics was revealed.

3. Supplementary materials for MD simulations

The conformational transformations leading to conformer S_0 substantially reshape the **MeBzS$_2$O** molecule, influencing the spatial distribution of its atomic charges. As a result, the emergence of conformer S_0 at lower temperatures significantly perturbs the arrangement of neighbouring molecules within its first coordination sphere. In order to dissect this phenomenon we analyze in details the spatial arrangement of molecules in models derived from MD simulations performed at 295 K. Figure S4 illustrates a random exemplary nearest surrounding of conformers S_0 and S_1. In contrast with the first coordination sphere of conformation S_1, we could easily find intermolecular distances less than 4 Å in the case of the nearest neighbourhood of conformer S_0 (marked by yellow lines in Figure S4a). These distances correspond to the

periodicities of structural correlations resulting in the appearance of the second peak in the diffraction pattern of **MeBzS$_2$O** positioned at Q~1.7 Å^{-1}. The prevalence of conformer S$_0$ increases significantly with lowering the temperature in the supercooled liquid regime, which corresponds to the considerable increase in intensity of the second diffraction peak below T_A (c.f. Figure 2e and Figure S3). The more rapid increase in the amplitude of the second diffraction peak emerges around the normal-to-supercooled liquid transition (i.e., around T_A), and correlates with the emergences of the conformer S$_0$ (this conformer does not survive above T_A). Consequently, it shows that the transformation occurring at this point goes far beyond molecular dynamics, being reflected also in the supramolecular architecture of the liquid.

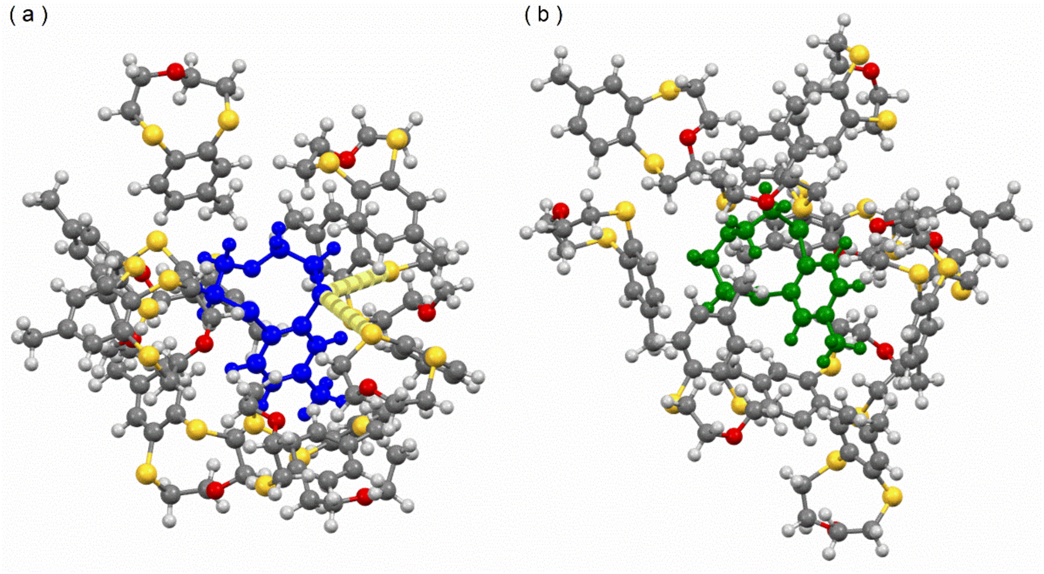

Figure S4. Selected fragments of MD structural models showing differences in the nearest neighbour of conformer S$_0$ (a) and conformer S$_1$ (b). The conformers S$_0$ and S$_1$ were marked in blue and green, respectively.

4. Additional data from DFT calculations

Table S1. DFT-optimized geometries of conformers S$_0$-S$_3$ defined by dihedral angles of the heterocyclic ring.

Dihedral angle φ	*Conformer S$_0$*	*Conformer S$_1$*	*Conformer S$_2$*	*Conformer S$_3$*
5-4-3-23	101.2^0	96.0^0	97.6^0	102.7^0
4-3-23-26	-87.3^0	-58.3^0	-107.8^0	-38.4^0
3-23-26-1	65.3^0	-59.1^0	48.5^0	-56.1^0
23-26-1-20	-140.7^0	152.0^0	60.1^0	137.8^0
26-1-20-17	140.0^0	-60.1^0	-152.0^0	-137.8^0
1-20-17-2	-64.8^0	-48.5^0	59.6^0	55.7^0
20-17-2-5	87.7^0	108.0^0	58.6^0	39.0^0
17-2-5-4	-102.1^0	-97.6^0	-95.9^0	-103.0^0

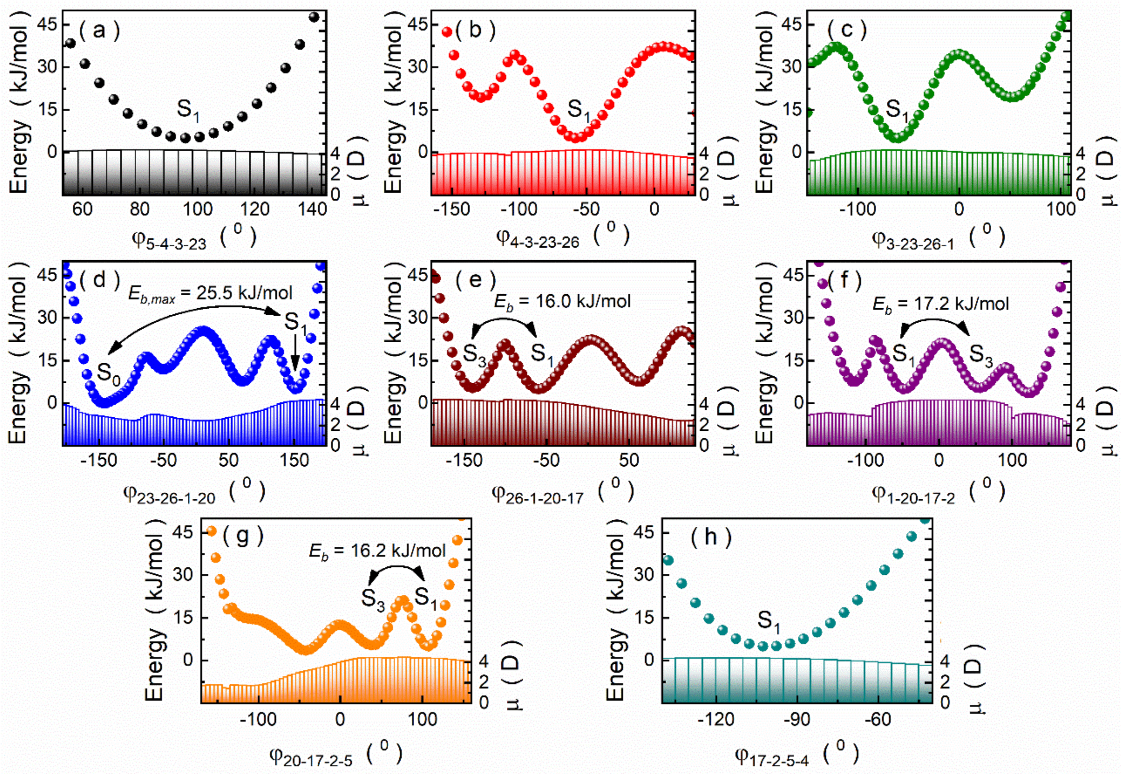

Figure S5. Changes in energy while altering the dihedral angles $\varphi_{5\text{-}4\text{-}3\text{-}23}$ (a), $\varphi_{4\text{-}3\text{-}23\text{-}26}$ (b), $\varphi_{3\text{-}23\text{-}26\text{-}1}$ (c), $\varphi_{23\text{-}26\text{-}1\text{-}20}$ (d), $\varphi_{26\text{-}1\text{-}20\text{-}17}$ (e), $\varphi_{1\text{-}20\text{-}17\text{-}2}$ (f), $\varphi_{20\text{-}17\text{-}2\text{-}5}$ (g), and $\varphi_{17\text{-}2\text{-}5\text{-}4}$ utilizing conformer S_1 as the initial geometry.

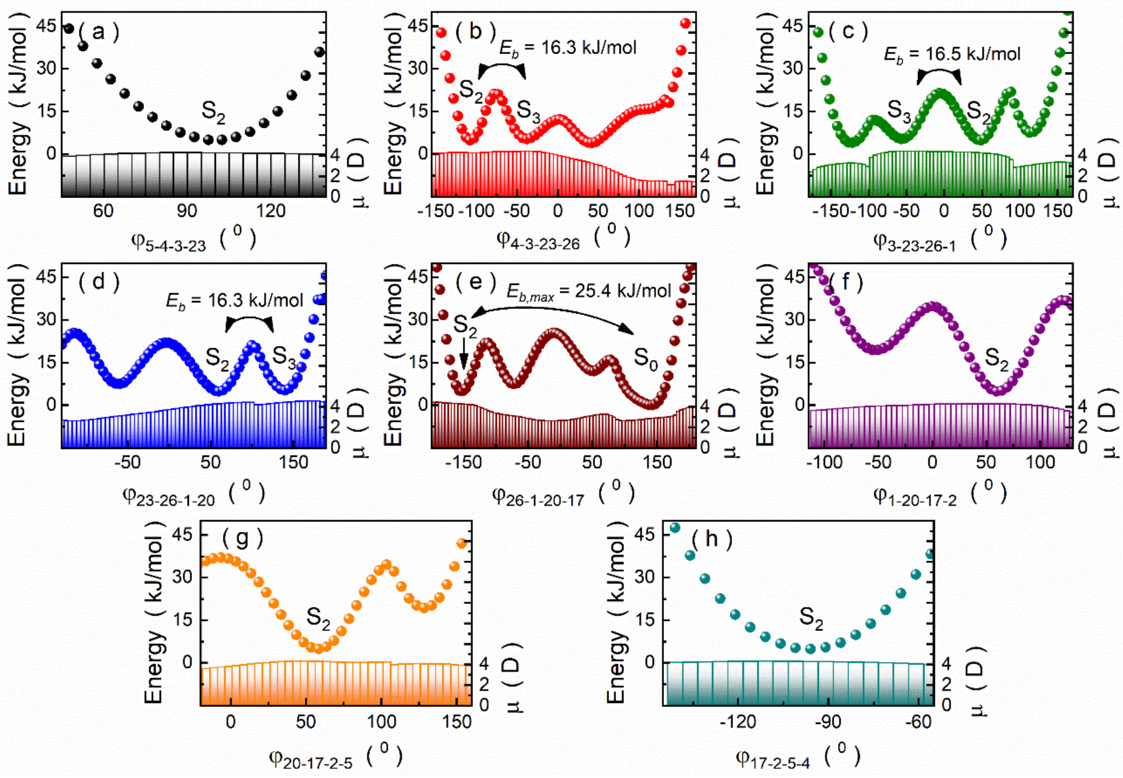

Figure S6. Changes in energy while altering the dihedral angles $\varphi_{5\text{-}4\text{-}3\text{-}23}$ (a), $\varphi_{4\text{-}3\text{-}23\text{-}26}$ (b), $\varphi_{3\text{-}23\text{-}26\text{-}1}$ (c), $\varphi_{23\text{-}26\text{-}1\text{-}20}$ (d), $\varphi_{26\text{-}1\text{-}20\text{-}17}$ (e), $\varphi_{1\text{-}20\text{-}17\text{-}2}$ (f), $\varphi_{20\text{-}17\text{-}2\text{-}5}$ (g), and $\varphi_{17\text{-}2\text{-}5\text{-}4}$ utilizing conformer S_2 as the initial geometry.

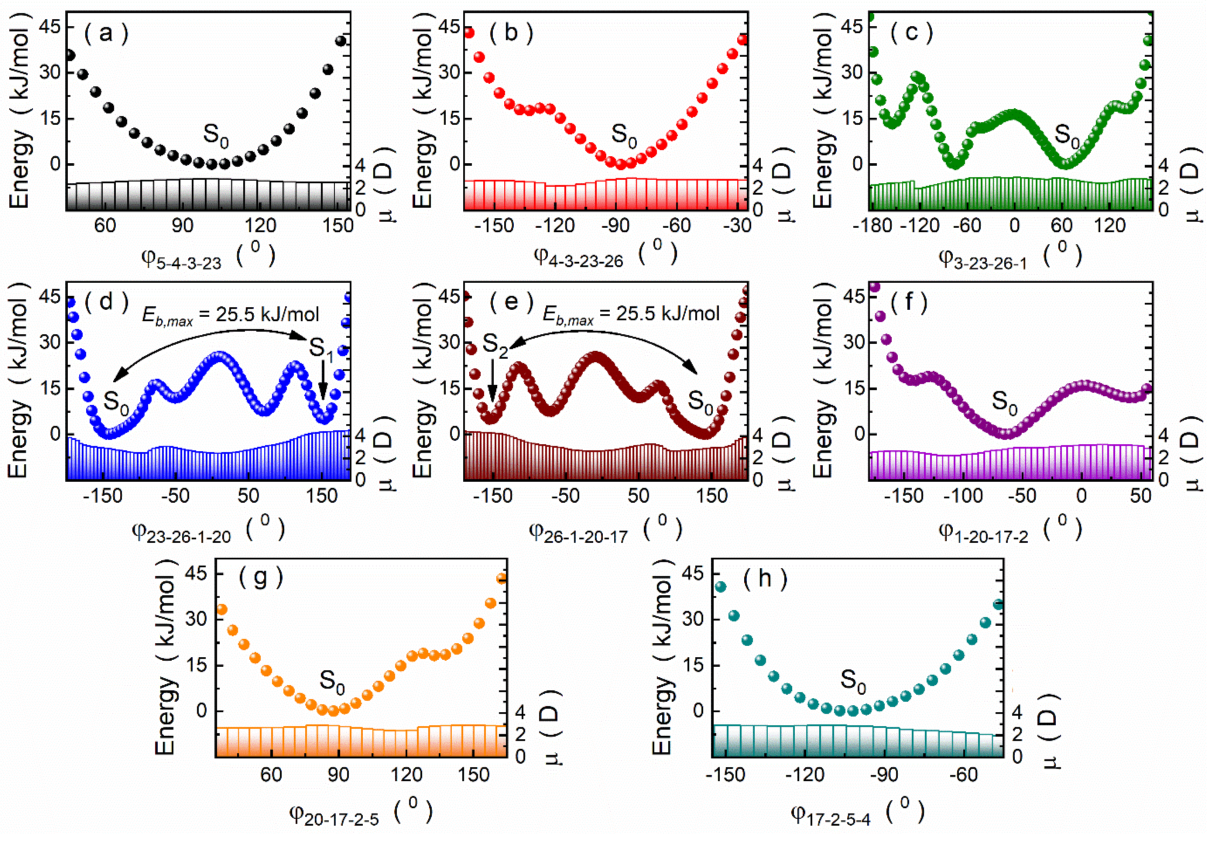

Figure S7. Changes in energy while altering the dihedral angles $\varphi_{5\text{-}4\text{-}3\text{-}23}$ (a), $\varphi_{4\text{-}3\text{-}23\text{-}26}$ (b), $\varphi_{3\text{-}23\text{-}26\text{-}1}$ (c), $\varphi_{23\text{-}26\text{-}1\text{-}20}$ (d), $\varphi_{26\text{-}1\text{-}20\text{-}17}$ (e), $\varphi_{1\text{-}20\text{-}17\text{-}2}$ (f), $\varphi_{20\text{-}17\text{-}2\text{-}5}$ (g), and $\varphi_{17\text{-}2\text{-}5\text{-}4}$ utilizing conformer S_0 as the initial geometry.

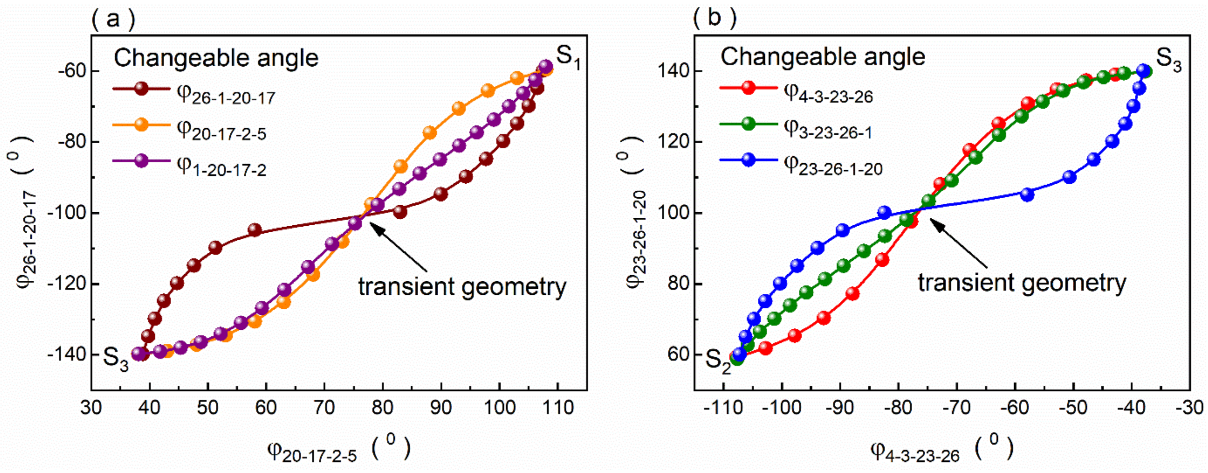

Figure S8. Different interconversion paths between conformers S_3 and S_1 (a), and conformers S_3 and S_2.

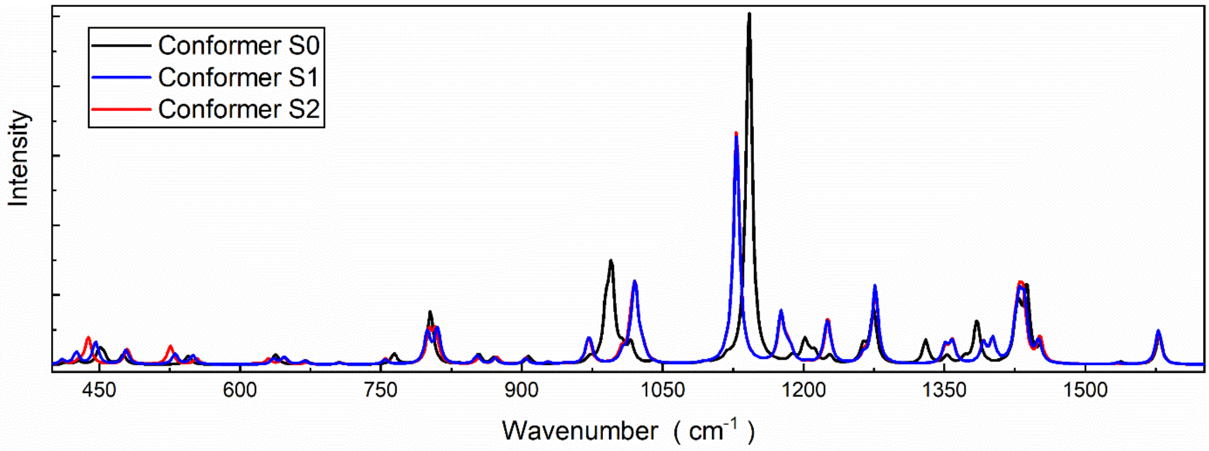

Figure S9. Simulated IR spectra of conformers S_0, S_1, and S_2 presented in the range of 400 – 1625 cm^{-1}.

IR spectra were simulated for conformers S_0, S_1, and S_2 at a hybrid B3PW91 level of theory and 6-311++G(d,p) basis set. The obtained results were scaled by vibrational frequency scaling factor proper for the selected method and basis, and presented in Figure S5. As can be seen, there are significant differences in the line shape among conformers S_0, S_1, S_2 between 400 and 1625 cm^{-1}. Consequently, consistent with MD simulations, we can ascribe the experimentally observed temperature-induced spectral changes to shifts in the prevalence of conformers coexisting with each other in the liquid phase.

5. References

[1] Cole, K. S.; Cole, R. H. Dispersion and Absorption in Dielectrics I. Alternating Current Characteristics. *J. Chem. Phys.* **1941**, *9*, 341–351.

[2] Havriliak, S.; Negami, S. A Complex Plane Representation of Dielectric and Mechanical Relaxation Processes in Some Polymers. *Polymer* **1967**, *8*, 161–210.

[3] Broadband Dielectric Spectroscopy; Kremer, F., Schönhals, A., *Eds.*; Springer Berlin Heidelberg: Berlin, Heidelberg, 2003

[4] Stickel, F.; Fischer, E. W.; Richert, R. Dynamics of glass-forming liquids. II. Detailed comparison of dielectric relaxation, dc-conductivity, and viscosity data. *J. Chem. Phys.* **1996**, *104*, 2043–2055.
